# Adaptive Fuzzy Logic Deep-Learning Equalizer for Mitigating Linear and Nonlinear Distortions in Underwater Visible Light Communication Systems

**DOI:** 10.3390/s23125418

**Published:** 2023-06-07

**Authors:** Radhakrishnan Rajalakshmi, Sivakumar Pothiraj, Miroslav Mahdal, Muniyandy Elangovan

**Affiliations:** 1 Department of Electronics and Communication Engineering, Ramco Institute of Technology, Rajapalayam 626117, India; 2 Department of Electronics and Communication Engineering, Kalasalingam Academy of Research and Education, Krishnankoil 626126, India; 3 Department of Control Systems and Instrumentation, Faculty of Mechanical Engineering, VSB-Technical University of Ostrava, 17. Listopadu 2172/15, 708 00 Ostrava, Czech Republic; 4 Department of R&D, Bond Marine Consultancy, London EC1V 2NX, UK

**Keywords:** underwater visible light communication, deep learning, equalization, adaptive fuzzy logic, deep-learning equalizer, sparrow search optimization

## Abstract

Underwater visible light communication (UVLC) has recently come to light as a viable wireless carrier for signal transmission in risky, uncharted, and delicate aquatic environments like seas. Despite the potential of UVLC as a green, clean, and safe alternative to conventional communication methods, it is challenged by significant signal attenuation and turbulent channel conditions compared to long-distance terrestrial communication. To address linear and nonlinear impairments in UVLC systems, this paper presents an adaptive fuzzy logic deep-learning equalizer (AFL-DLE) for 64 Quadrature Amplitude Modulation-Component minimal Amplitude Phase shift (QAM-CAP)-modulated UVLC systems. The proposed AFL-DLE is dependent on complex-valued neural networks and constellation partitioning schemes and utilizes the Enhanced Chaotic Sparrow Search Optimization Algorithm (ECSSOA) to improve overall system performance. Experimental outcomes demonstrate that the suggested equalizer achieves significant reductions in bit error rate (55%), distortion rate (45%), computational complexity (48%), and computation cost (75%) while maintaining a high transmission rate (99%). This approach enables the development of high-speed UVLC systems capable of processing data online, thereby advancing state-of-the-art underwater communication.

## 1. Introduction

Underwater visible light communication (UVLC) is an intensity modulation method accessible by light-emitting diodes (LEDs). Adjusting the LED’s light wave outcome may accelerate rapid data transfer speeds. Future networking systems have a lot of potential for this technology. UVLC will soon get closer to flexible connectivity (FC), enabling it to continuously and creatively deploy energy to meet the demands of different frequency bands and improve spectrum utilization.

Frequent significant FC technologies are “Modulation Classification (MC) technologies”. This refers to a piece of information technology that is frequently utilized and capable of autonomous learning and adaptation. MC may help the transmitter correctly identify and change the demodulation modes when the broadcast protocols are altered due to external conditions. Real-time functioning is, therefore, a necessity for MC [1]. The creative improvement of UVLC, in line with the 5 G and 5 GB goals for future wireless communications, will increase operational effectiveness globally while still satisfying the demands of the moment. With minimal eavesdropping, UVLC offers extremely safe data transmission. A downlink data speed of more than 1 Gbps is provided by 5 GB, a sophisticated wireless connectivity messaging technique. However, a drawback of 5GB is the high expense of building infrastructures.

However, the current transfer of VLC by the 6G connection network, as opposed to the 5G network, is more pronounced in terms of augmented machine transmission and hyper-reduced latency communications. RF transmission technologies experience interference, but functional VLC technologies are unaffected. Therefore, VLC may be utilized with significant benefits in industries including the medical field, the oil and gas business, “nuclear power plants (NPPs)”, underwater interactions, and many more uses where RF wireless communication is not commonly employed [2].

Recent years have seen increased demand for high-speed underwater wireless communication (UWC) techniques due to underwater use cases, including underwater vehicle monitoring, seabed geomorphology, and seawater cleanliness monitoring [3]. Underwater acoustic networking is a well-established method; however, due to its constrained frequency, it has a limited data capacity. Due to radio’s energy absorption in saltwater, radio frequencies are not suited for underwater communications. Due to blue and green light’s ability to pass through water with comparatively minimal absorption, UVLC, which relies on blue and green semiconductor lasers and LEDs, offers high-performance and affordable UWC solutions [4,5].

**Figure 1:**
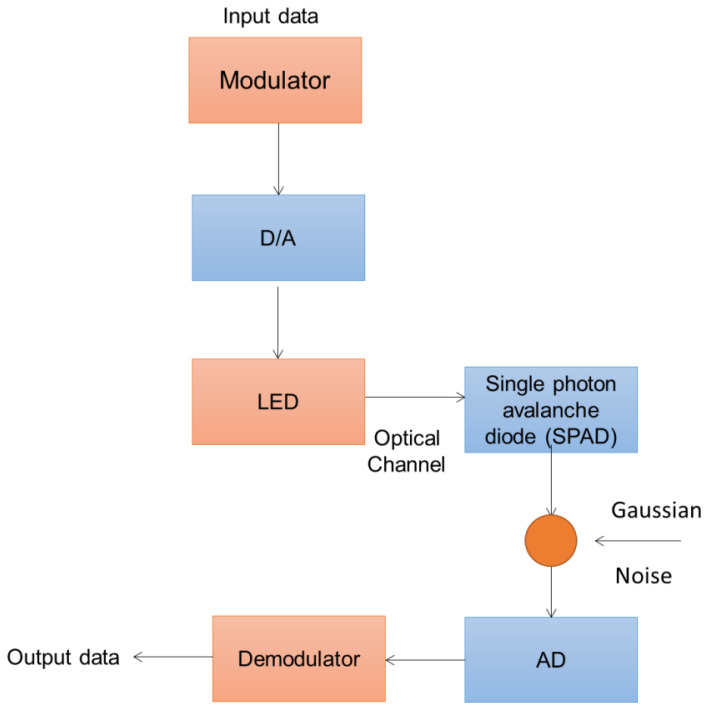
General block diagram of UVLC system model.

The system model for UVLC features a block architecture with a single photon avalanche diode (SPAD) receiver and one source light-emitting diode (LED). The main advantages of using LED devices for underwater transmission include their fast switching times, excellent illumination, affordability, low electric power consumption, high moisture resistance, and high-speed data transmission capabilities. Figure 1 depicts the general block diagram of the UVLC system model, which employs a single source LED and a single SPAD receiver [6]. The SPAD is used in long-distance VLC systems because of its high sensitivity and excellent detection efficiency. The SPAD is specifically made to operate with a reverse bias voltage substantially larger than the breakdown voltage, as opposed to the avalanche photodiode (APD), which performs with a bias greater than the breakdown voltage.

“D/A expression” may define the steps involved in decoding (D) and encoding (A) the UVLC codes. While the encoding phase involves transforming the symbols or data items into UVLC codes for transmission, the decoding step entails translating the received UVLC codes back into their original symbols or data elements. Investigators are looking into better modulation methods for UVLC devices to boost transmission rates. The advantages of “component minimum amplitude phase shift (CAP) with M-order quadrature amplitude modulation (M-QAM)”, including good spectrum effectiveness and a low peak-to-average ratio, are frequently cited. Yet, linear and nonlinear impairments are unavoidable in a true high-speed UVLC system, significantly lowering the possible transmission rate and system efficiency. These limitations are mostly the outcome of defects in optical components, including lighting, electrical amplifying systems, photosensitive elements, and the complicated underwater environment that results in dispersion, diffraction, and turbulence. Post-equalizers have been created for UVLC systems to address these shortcomings successfully. The least mean square (LMS) and Volterra series approaches are common equalizer methods. Volterra is frequently used for nonlinear normalization, while LMS concentrates on linear compensation [7].

Neural networks (NN) have gained significant interest, especially in optical communication [8]. NNs have been demonstrated to be highly effective in approximating complex functions by learning from large datasets. Due to their capability, NNs are a promising option for mitigating nonlinear distortion [9,10]. Greater nonlinear distortion is observed in the UVLC system for higher power messages compared to lower power signals. In previous research, random sequential data was handled using long short-term memory (LSTM) networks that depend on machine learning approaches. A total of 13.5 Gbps of data speed and a 2-m connection distance, these networks have been utilized to demonstrate a high-speed VLC connection. The collected M-QAM signals show various distortion levels at various symbol strengths, mainly when applying high-order modulation. The usual configuration shows that symbols in the outer part of the constellations are more prone to nonlinear impairments than those in the inner portion. The UVLC system is most affected by distortions, which increases its complexity and effectiveness. We suggest the idea of an adaptive fuzzy logic deep-learning equalizer to address this problem and reduce these drawbacks.

The structure of essay is as follows: Section 2 indicates a literature review concerning the proposed work; Section 3 provides a detailed study of the suggested novel HC framework; Section 4 analyzes the comparative and experimental results of the proposed work; and lastly, Section 5 indicates the study and describes future work.

## 2. Literature Survey

Niu et al. [11] demonstrated the effectiveness of a “Super Gaussian kernel assisted deep neural network (SGK-DNN)” equalization in “blue LED CAP 16QAM visible light communication”, spanning from 10 cm of open space to a 100 m cable. Although SGK-DNN equalization efficiently mitigates highly nonlinear distortion, it has higher computational complexity. Cai et al. [12] introduced a post-equalization approach based on Bidirectional Gated Recurrent Units (BGRU) for mitigating linear and nonlinear distortion in underwater visible light communication using precise mathematical programming and structure amplitude phase shift keying. However, this approach consumes more energy during operation.

Jin et al. [13] proposed a “Functional Link Neural Network” as a post-equalization technique in underwater visible light communication technology for nonlinear equalization. Du et al. [14] presented an in-depth analysis of intelligent hardware and network layer techniques for an AI-enabled advanced UVLC system. They discussed the issues arising from nonlinear distortions and potential solutions. They also suggested and empirically demonstrated an innovative “fully-connected deep neural network (FC-DNN)” based transmitter in an “underwater wireless optical communication (UWOC) system to directly decode orthogonal frequency division multiplexing (OFDM)” data using deep learning techniques [14].

Niu et al. [15] advocated for using a support vector machine (SVM), which has been extensively studied in quadruple frequency modulation for decryption to make softer decisions, manage to increase computational complexity, and eliminate distortion. Li et al. [16] suggested using a gated recurrent unit (GRU) neural network-driven equalization to compensate for linear and nonlinear distortions in carrier-less amplitude-phase band-constrained UVLC systems. The GRU method is the primary basis of the equalization technique, using a gated approach to capture the relationships between segments stored within the system.

In their study, Lu et al. [17] designed an optoelectronic convolutional neural network (OECNN) for data processing equalization based on an optoelectronic multilayer processing unit. This enabled them to recover information in optical transmission channels while reducing linear and nonlinear distortions and lowering the substantial computing expense. The optical transmission information restoration system is founded on an optical convolution decoder implemented using a propagation delay module and frequency-division multiplexer.

Zhao et al. [18] suggested using transmission learning-depends on artificial neural networks as subsequent equalizers for UVLC to mitigate linear and nonlinear distortion while reducing computational complexity. Chen et al. [19] developed a novel nonlinear robust learning post-equalization for UVLC systems in their research. The proposed “Time-frequency domain network (TFDNet)” leverages time-frequency pixel analysis, simultaneously considering both time and frequency contexts. This converts the signal into a 2D feature, which a neural system then learns to address nonlinear imbalances in UVLC. Conventional equalizers only account for the time domain.

Zhao et al. [20] demonstrated a new post-equalizer dependent on a “dual branch multi-layer perceptron (DBMLP)”. The dual branching of the DBMLP compensates for linear and nonlinear distortions in the received data, reducing the difficulty of the artificial system and improving the post-equalizer’s effectiveness. Zhao and Chi [21] implemented a partial reduction technique for post-adjustment dependent on a “dual-branch multilayer perceptron-based post-equalizer (DBMLP-PE)” in a UVLC system to remove distortions and significantly reduce computational cost.

Shen et al. [22] recommended and experimentally verified a high-speed and robust UVLC technology utilizing a single input multi-output oriented adaptive diversity combining technique for efficiency improvements. To combat underwater interference, the Maximal Ratio Combining method is used for the receiving end, while the multi-layer perceptron (MLP) technique is utilized to mitigate nonlinear deficiencies. The deep neural network (DNN) outperforms the least mean square equalizer. A digital low-pass filter and a high-pass filter are then used to separate the received signal into two parallel streams. However, excessive nodes make the NN more complex, and the update process only partially mitigates distortion [23].

The significant nonlinear distortions of underwater PAM8 VLC channels may be effectively compensated for by a “Gaussian kernel-aided deep neural network (GK-DNN) equalizer” [24]. Incorporating categorical variables is challenging, as Gaussian mixture models need to be better suited to categorical data, assuming all features are typically distributed. The performance of deep learning (DL) methods for AMC in VLC systems is empirically examined [25]. Yet, the majority of active learning (AL) methods for VLC systems rely on AMC techniques that necessitate a substantial amount of labeled training information that is challenging to get in practical systems.

Article [26] examined the effectiveness of the suggested ocean monitoring system, which links marine and terrestrial life. The communication system is supported by a satellite link for constant real-time monitoring and all-encompassing coverage. Underwater visible light communication (UVLC) is used to transfer sensor data from several sensor nodes (SN) positioned at various depths to underwater vehicles (UV). For a single chip silicon-substrate light-emitting diode (LED) based underwater visible light communication (UVLC), we build a real-time discrete multi-tone (DMT) transceiver based on FPGA chips [27].

## 3. Problem Statement

Due to distortions brought on by the intricate underwater channel and the nonlinear properties of the light source, underwater visible light communication (UVLC) devices encounter substantial difficulties. When utilizing modern modulation techniques to achieve high transmission speeds, these distortions might reduce the quality of the information received and create problems. An adaptive fuzzy logic deep-learning equalizer can be suggested as an answer to these problems in order to correct for the distortions in UVLC systems. In conclusion, the suggested adaptive fuzzy logic deep-learning equalizer offers a potentially effective way to reduce distortions in UVLC systems. The equalizer corrects for distortions, boosts performance, scales down in complexity, and adjusts to shifting undersea conditions, thereby improving UVLC communications’ dependability and effectiveness. It does this by combining adaptive filtering, fuzzy logic, and deep learning approaches.

## 4. Proposed Framework

We introduce an adaptive fuzzy logic deep-learning equalizer (AFL-DLE) for 64 QAM-CAP modulated UVLC systems dependent on complex-valued neural networks and a constellation partitioning algorithm to overwhelm the linear and nonlinear imperfections in UVLC. The system’s efficiency is improved by utilizing the Enhanced chaotic sparrow search optimization algorithm (ECSSOA). The audio signal features via a training data set can provide precise classification boundaries. I/Q components, which stand for in-phase and quadrature, are often concatenated. The kernel function can transform information from a “lower-dimensional feature space to a higher-dimensional feature space”, allowing for linear separation of the training data set if it cannot be linearly separated [15].

### 4.1. Adaptive Fuzzy Logic Deep-Learning Equalizer (AFL-DLE)

**Figure 2:**
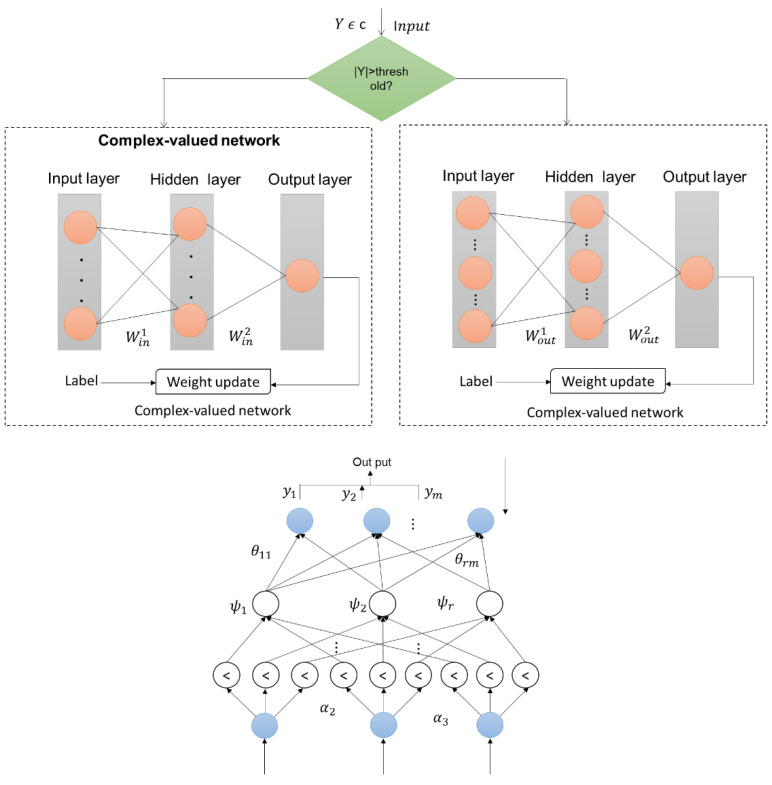
The architectural structure of the proposed AFL-DLE.

Due to the incoherent properties of LEDs, amplitude regulation and direct sensing approaches are used in UVLC systems that employ LEDs. A high motoring bias is necessary to increase the “signal-to-noise ratio (*SNR*)” due to the important amplification of the water phase. This induces nonlinear disturbance arising from both the underwater environment and spatial hardware responses, including the variational electro-optic transformation of LEDs, the nonlinear reaction of electronic systems, and the square-law detection of photodiodes. The UVLC system has a nonlinear reply to the standardized intensity amplitude slope of the input signal and output signal (a). As the signal’s intensity increases, the nonlinearity becomes more noticeable, representing that the received symbols’ degrees of distortion vary. The received symbols are normalized by the LMS procedure for an intuitive demonstration. In the subsequent LMS equalization, the green and black spots denote the proper and improper symbols, respectively. The majority of black spots are scattered in the constellation’s exterior, suggesting that LMS has insufficient equalization capability to handle received patterns with greater imperfections located there. Therefore, we introduce an adaptable segmented equalization to equalize the affected components with increased effectiveness and reduced complexity. We employ neural networks to address nonlinear distortion. Figure 2 presents the architectural structure of the proposed AFL-DLE.

The obtained M-QAM message series is represented as $Y=\left[y_{1},y_{2},\ldots ,y_{n}\right]$, where the vector $y_{l}\left(l=1,2,\ldots ,n\right)$ denotes the lth obtained message. Symbols and every vector $y_{l}$ have difficult values, containing the in-phase (I) and quadrature-phase (Q) components, and are located in the complex domain C. The actual transmitted pattern is designated as $X=[{\hat{x}}_{1},{\hat{x}}_{2},\ldots ,{\hat{x}}_{n}$], while the matching anticipated sequence is designated as $X=[{\hat{x}}_{1},{\hat{x}}_{2},\ldots ,{\hat{x}}_{n}$]. Normalization aims to attempt to retrieve the expected X from Y that closely resembles the actual X. The obtained symbols $Y=\left[y_{1},y_{2},\ldots ,y_{n}\right]$ are divided into two divisions by a cutoff first, with $Y_{\mathrm{inner}}$ located in the inner area of the cluster and $Y_{\mathrm{outer}}$ located in the outer portion.

As seen in Figure 2, the threshold’s border has a rectangle form. The amplitude of acquired signs must be considered when using other forms, such as the round. This research divides the signs into rectangles based on I/Q signs for simplification and as proof of concept. Finding signs whose magnitude of real and imaginary portions are inside the threshold border is the first step in the split procedure for $Y_{\mathrm{inner}}$, after which $Y_{\mathrm{outer}}$ is responsible for the remaining symbols. Decreased computing difficulty while preserving acceptable equalization effectiveness depends on selecting the appropriate cutoff. We provide a flexible method for determining the best threshold depending on the bit error rate (*BER*) after LMS normalization. Then, two complex-valued neural networks equalize $Y_{\mathrm{inner}}$ and $Y_{\mathrm{outer}}$ correspondingly. We employ the FCNN design, which consists of a single intake layer, one concealed layer, and one output layer, for the complicated exponential neural network. The incoming signal, $Y_{\mathrm{inner}}$ (*i*), propagates as complex values during the concealed layer using $L_{\mathrm{inner}}$ symbols positioned on the middle symbol. Equation (1) represents how the network’s complicated output is stated.

(1) $${\hat{x}}_{inner}=W_{in}^{2}\left(f\left(W_{in}^{1}Y_{\mathrm{inner}}+B_{in}^{1}\right)\right)+B_{in}^{2}$$

The weighted matrices and biased vectors with complex elements are $W_{in}^{j}$ and $B_{in}^{j}\left(j=1,2\right)$. The activating factor is shown by the symbol *f* (·). The excellent efficiency of the ReLU activating mechanism led to its selection. Equation (2)’s expression for the complexity ReLU activating factor, which assigns distinct ReLUs to neuron *z*’s real and imaginary parts, is what we utilize.

(2) $$\mathbb{C}ReLU\left(z\right)=ReLU\left(Re\left(z\right)\right)+iReLU\left(im\left(z\right)\right)$$

In Equation (3), the mean square loss provides the complicated network’s loss rate.

(3) $$E=\frac{1}{M}\overset{M}{\underset{m=1}{\sum }}e_{inner}{\tilde{e}}_{inner}$$

The network values and biases are then adjusted using the suggested method. The sectioned equalization is prepared to normalize the input signs that still need to be seen after the training processes for $Y_{\mathrm{inner}}$ and $Y_{\mathrm{outer}}$ have been completed.

Numerous traditional approaches fall short of expectations because they rely so much on precise statistical assumptions. Because of the inconsistencies and large deformations present in reality, it is not simple to derive an exact solution. Fortunately, these restrictions are not current with soft computing techniques since they are not dependent on a formal model, allowing for faster speed. In practice, these techniques are primarily used in several situations where the accuracy of statistical equations cannot be assured owing to massive inputs with high noise levels and continually shifting characteristics. Figure 2 depicts the adaptive fuzzy logic design. It has four levels: layers 1 and 2 make up the fuzzy rules’ anterior portion and are made up of the inputs and fuzzification nodes, accordingly; layers 3 and 4 make up the fuzzy rules’ subsequent portion and are made up of the fuzzy regulation and outcome nodes, accordingly. The variable matrix connects these layers. Any well-described nonlinear function may be consistently approximated by fuzzy logic over a limited set U to any level of precision.

A participation mechanism named A that assigns each component of the discussion language X a participation score named A(l) in the range [0, 1] may be used to construct fuzzy series. As a result, the fuzzifier step entails translating a sharp input X into a fuzzy output A. The inferred mechanism is crucial for any fuzzy units because it combines the fuzzy values supplied by the fuzzification step with the information base IF-THEN rules to build a complete result fuzzy set.

When paired with the fuzzified inputs, every rule in the information base is seen as having a fuzzy consequence, and Equation (4) defines the fuzzy set Bl as a result.

(4) $$\mu_{B}{}^{l}\left(\beta \right)={⊔}_{\alpha \in }X\left[\mu A_{\alpha }\left(\alpha \right)⊓R^{l}\left(\alpha ,\beta \right)\right]$$

A phase of defuzzification is required before the sharp factor of the finalized result can be calculated. For fuzzy logic, various defuzzification strategies are available, such as the centroid and center-of-sums defuzzifiers. The result at the moment *k* may be represented as the following Equation (5) if a defuzzification technique that is routinely used, such as centroid, is utilized.

(5) $$yj^{\left(k\right)}=\frac{\sum_{l=1}^{r}\theta_{l}\mu B^{l}\left(\beta \right)}{\sum_{l=1}^{r}\mu B^{l}\left(\beta \right)}$$

### 4.2. Optimization Using Enhanced Chaotic Sparrow Search Optimization Algorithm (ECSSOA)

The Enhanced chaotic sparrow search algorithm (ECSSOA) is a kind of smart program that has been used in several sectors because of its distinctive qualities, including a strong capacity to do global searches, a limited number of programmable variables, and a concise architecture [28].

The density matrices are illustrated in Equation (6), determining that there are *N* sparrows in the D-dimensional area (1).

(6) $$X={\left[x_{1},x_{2},\ldots x_{N}\right]}^{T},x_{i}=\left[x_{i}1,x_{i},2,\ldots x_{i}D\right]$$

where $x_{i}$, *D* denotes the *i*th sparrow’s location in dimension *D*.

Operations generally comprise 10% to 20% of the total populace, and Equation (7) updates the position.

(7) $$x_{i,j}^{t+1}=\left\{\begin{array}{c} x_{i,j}^{t}exp\left(\frac{-i}{\alpha \cdot iter_{max}}\right)A_{2}<CD\\ x_{i,j}^{t}+Q\cdot LA\geq CD \end{array}\right.$$

where t is the present iteration count. j=1, 2, ···, d. The highest number of repetitions is represented by itermax. α is a deterministic randomized number between 0 and 1. The warning and security levels for sparrows are represented by $A2\;\left(A2\;\in \;\left[0,\;1\right]\right)$ and $CD\;\left(CD\;\in \;\left[0.5,\;1.0\right]\right)$, correspondingly. The randomized number Q has a regular dispersion, as expected.

Each member of the matrix L, which has the dimensions d, is 1. The product enters a wide-area searching mode when A2<CD signals that there are no biological competitors nearby and that the region is mainly secure. If A2>CD indicates that the producer is conscious of a biological competitor present, then the producer must move to a different region to feed. Equation (8) causes an update to the follower location.

(8) $$x_{i,j}^{t+1}=\left\{\begin{array}{c} Q\cdot exp\left(\frac{x_{worst}^{t}-x_{i,j}^{t}}{i^{2}}\right)i>\frac{n}{2}\\ x_{p}^{t+1}+\left|x_{i,j}^{t}-x_{p}^{t+1}\right|\cdot A^{+}\cdot Lotherwise \end{array}\right.$$

where $x_{worst}^{t}$ denotes the location of the sparrow with the poorest adaptation. The number xp represents the location of the sparrow with the best production adaptation. Each element of the matrix represented by the letter *A* is given a randomized value of one or zero. $A+=AT\;\left(AAT\right)-1$. In addition, $i>\frac{n}{2}$ indicates that the ith sparrow is probably starving and must go to a different place to graze.

Chaos is a chaotic phenomenon that occurs naturally and has been used to improve computations [29,30]. Due to its chaotic and harmonic properties, it enhances the variety of populaces and makes it easier for the program to depart from the locally optimal. A common chaotic transfer is a cubic translation, and Equation (9) illustrates its standardized format.

(9) $$x_{n+1}=bx_{n}^{3}-cx_{n}$$

where the chaotic effect factors are b and c. The series produced by the cubic mapping is the chaotic series for c (2.3, 3). The highest Lyapunov exponents were calculated for 16 typical chaotic maps, including the Cube map, and the Quadratic map-based equation was modified. According to the research findings, a Cubic map has less chaotic effect than one-dimensional maps like Sine mapping and Circle mapping but is superior to those worm face maps and tented maps. Equation (10) shows the adjusted Cube map the ECSSOA uses for initialization of the inhabitant’s solution.

(10) $$x_{n+1}=\rho x_{n}\left(1-x_{n}^{2}\right)$$

where ρ is the control variable.

A higher persistence value is required in the initial repetitions to extend the discoverer’s worldwide searching area since the production undertakes worldwide research as rapidly feasible in the first repeats to identify the worldwide ideal option swiftly. Simultaneously, a reduced persistence value is required in the final repetitions to enhance the discoverer’s localized extraction capabilities, in addition to accelerating divergence and preventing settling on the local optimum answer. Hence, adaptable values present a unique enhancement to the production location enhancement updating Equation (7), and the production location formula is represented in Equation (11).

(11) $$x_{i,j}^{t+1}=\left\{\begin{array}{c} \omega \cdot_{i,j}^{t}exp\left(\frac{-i}{\alpha \cdot iter_{max}}\right)A_{2}<CD\\ \omega \cdot x_{i,j}^{t}+Q\cdot LA_{2}\geq CD \end{array}\right.$$

The precise computation of ω is expressed in Equation (12).

(12) $$\omega =\left\{\begin{array}{c} \omega_{0}t\leq t_{0}\\ {\left(\frac{1}{t}\right)}^{0.9}t\leq t_{0} \end{array}\right.$$

where 0 represents the affirmative real number that is provided. The number of repetitions is indicated by t. The specified range of repetitions is $t_{0}$. In the sparrow searching phase, the system increases the scale factor in the initial iteration to increase the general searching area and the scale factor in the later repetition to increase the localized extortion capabilities.

**Figure 3:**
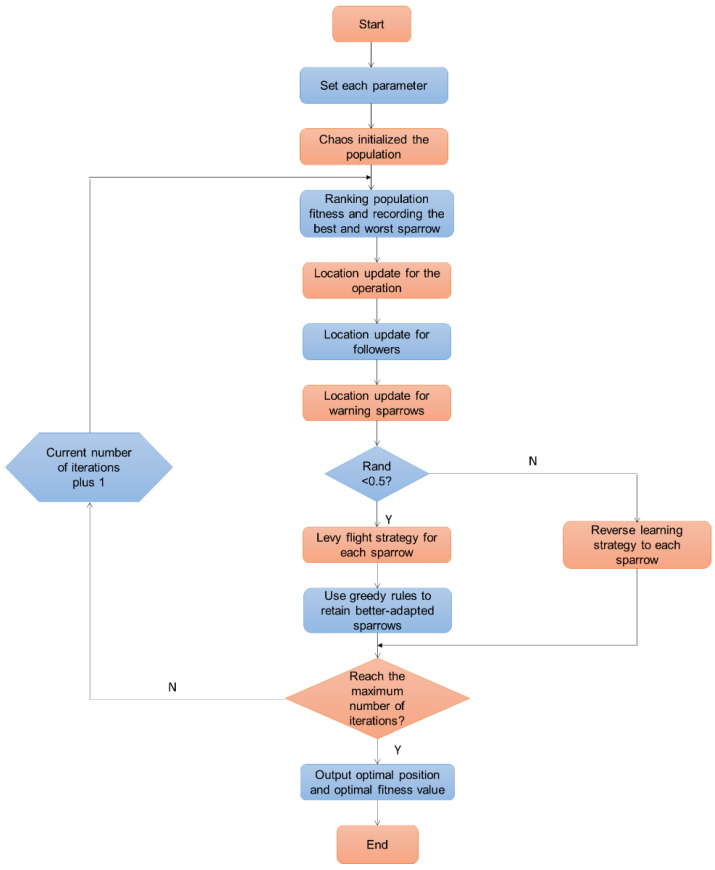
Flow chart of ECSSOA.

Figure 3 shows the flow chart of ECSSOA. A class of randomized non-Gaussian phenomena is called Levy flight. A high spotted probability of scatter plots best describes the probabilistic model of rise time. Levy flight can enable optimization techniques that are susceptible to running into the problem of the localized optimal to leap out of the area perfectly by a huge step with a greater chance of occurring in the stochastic process [31,32]. Each sparrow location’s Levy flying plan will be revised utilizing the greedy regulations to maintain the advantageous location. Reverse learning is a technique used to identify the inverse response corresponding to the present solution and keep the better alternative after assessment. Since backward training can locate the superior answer, programs often employ it to leave the optimum solution. Reverse learning was used to update the sparrow’s location. When most iteration has been completed, it offers the precise location and accurate solution.

### 4.3. Experimental Configuration

**Figure 4:**
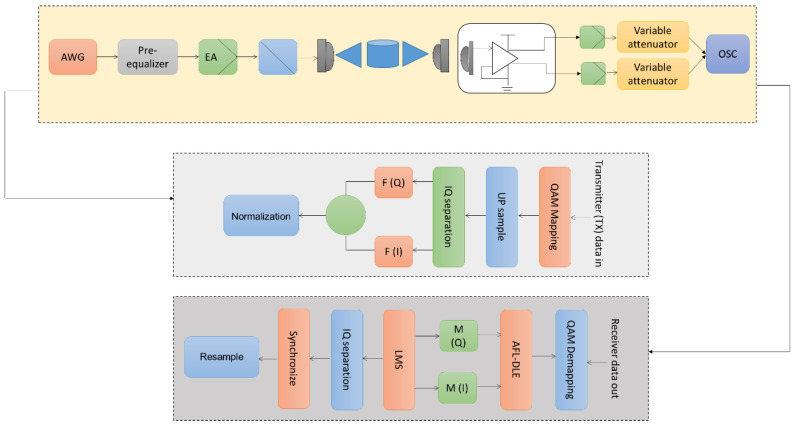
Experimental configuration.

The experimental configuration for the 64 QAM-CAP-regulated UVLC technologies, which comprises the underwater path, the transmitters, and the receiver, is depicted in Figure 4. A MATLAB application produces the initial digital data. To implement deep learning, predictive modeling, and statistical analysis methods, research groups use MATLAB 2022. Data for measured input and output are specified for an experiment. During the estimate, the model is simulated using the experiment input data, and the model output is contrasted with the actual experiment output data. The initial bit pattern is first translated into 64 QAM codes at the broadcaster using QAM mapping. The pattern is submitted for CAP modulated utilizing the cube-raised cosine function after four iterations of up-sampling. The random wave generation (Tektronix AWG 710B, Beaverton, OR, USA) is then fed with the standardized pattern to produce the analog electronic current. A hardware pre-equalization circuit prequalifies the electrical current, amplified by an electrical amplifier and linked with a bias tee to drive a Si-based LED with a silicon substrate. A lens then concentrates the photons so they may pass through the water tank. Due to technical restrictions, a 1.2 m water tank is employed as the underwater route. It should be emphasized that this research uses static water; further study will consider additional underwater pathway disruptions and greater transmission distances. An offerings PIN photodiode (Hamamatsu 10784, Bridgewater, NJ, USA) is utilized at the transmitter to employ differential power amplifier circuitry to transform the light brightness into an electronic current. A data memory analyzer then samples the incoming message impulses. The sinusoidal signal patterns first proceed through synchronization for the DSP on the receiving device. The linear distortion of the incoming signal is partially equalized using LMS. The QAM signs are acquired after the corresponding filters and down-sampling, and the suggested AFL-DLE equalizer processes them next. Here, evaluations are also made with other equalizers. The bit error rate (*BER*) is computed after 64 QAM demapping to assess system effectiveness.

## 5. Results and Discussion

This section evaluates the proposed system’s effectiveness in eliminating linear and nonlinear distortions in the UVLC system. Bit error rate, distortion rate, computational complexity, transmission rate, and computation cost are the key metrics to evaluate the efficiency of the proposed AFL-DLE performance. These metrics were contrasted with existing techniques for efficiency comparison. The conventional methods are Adaptive partitioning neural network (APNN), temporal convolutional neural network (TFCNN), Dual Self-Attention Network (DSANet), and “convolution-enhanced long short-term memory (CE-LSTM)”.

### 5.1. Bit Error Rate

**Figure 5:**
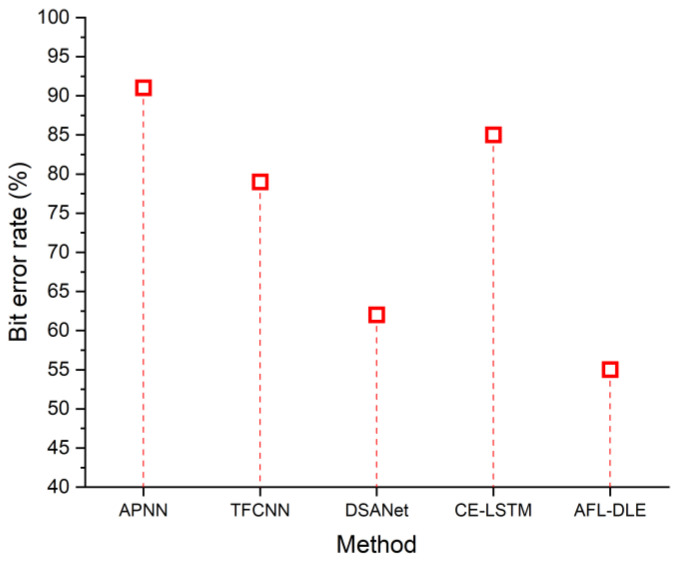
The bit error rate of the proposed and existing techniques, namely APNN [33], TFCNN [34], DSANet [35] and CE-LSTM [36].

When the threshold rises over a certain level, *BER* dramatically rises. This indicates that a more potent equalization should be employed since the approach can no longer adequately correct the deficiencies. Thus, the threshold is decided to be the mutational origin of *BER*. It is important to note that the ideal threshold changes as the data rate varies. The *BER* rate similarly decreases as the threshold dose. As a result, the distortions may be eliminated effectively. The bit error rates of the existing and suggested approaches are depicted in Figure 5. Table 1 indicates the contrast of the bit error rate (%). It demonstrates that the proposed method has a 55% lower bit error rate. As a result, it removes the distortions in the UVLC system. In VLC, information is transmitted by changing the visible light spectrum utilized for lighting. Through analytical and experimental studies, it has been shown that VLC can offer high-speed data transmission with the additional advantage of increased energy efficiency and communication security. The formula for *BER* is denoted as follows:

(13) $$BER=\frac{1}{2}erfc\left(\sqrt{E_{b}/N_{0}}\right)$$

The *BER* is often referred to as a function of the normalized carrier-to-noise ratio measure represented as *E_b_*/*N*_0_.

**Table 1:** Comparison of bit error rate (%).

Method	Bit Error Rate (%)
APNN [33]	91
TFCNN [34]	79
DSANet [35]	62
CE-LSTM [36]	85
AFL-DLE [Proposed]	55

### 5.2. Distortion Rate

**Figure 6:**
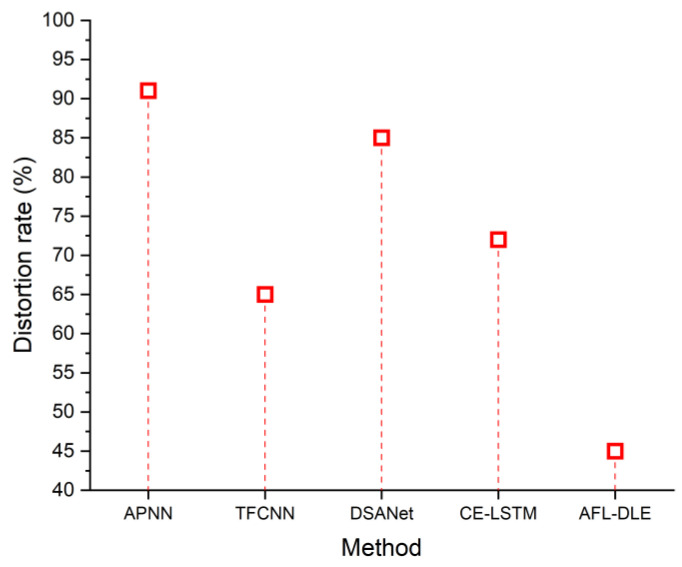
Distortion rate of the proposed and existing techniques, namely APNN [33], TFCNN [34], DSANet [35] and CE-LSTM [36].

The distortion rate indicates the amount of distortion in the UVLC system. The technique and how effectively it eliminates the linear and nonlinear distortion in the UVLC system is essential for efficient transmission. Figure 6 displays the distortion rate for both the existing and recommended methods. Table 2 shows the comparison of the distortion rate (%). The suggested system’s distortion rate is minimal, at 45%. It means that the recommended strategy eliminates UVLC system distortions. It may be computed utilizing the following formula and is commonly expressed in decibels (dB):

(14) $$SNR=10*log10\left(P_{s}/P_{d}\right)$$

$P_{s}$ denotes the power of the signal.

$P_{d}$ denotes the power of the noise or distortion.

**Table 2:** Comparison of distortion rate (%).

Source	Distortion Rate (%)
APNN [33]	91
TFCNN [34]	65
DSANet [35]	85
CE-LSTM [36]	72
AFL-DLE [Proposed]	45

### 5.3. Computational Complexity

**Figure 7:**
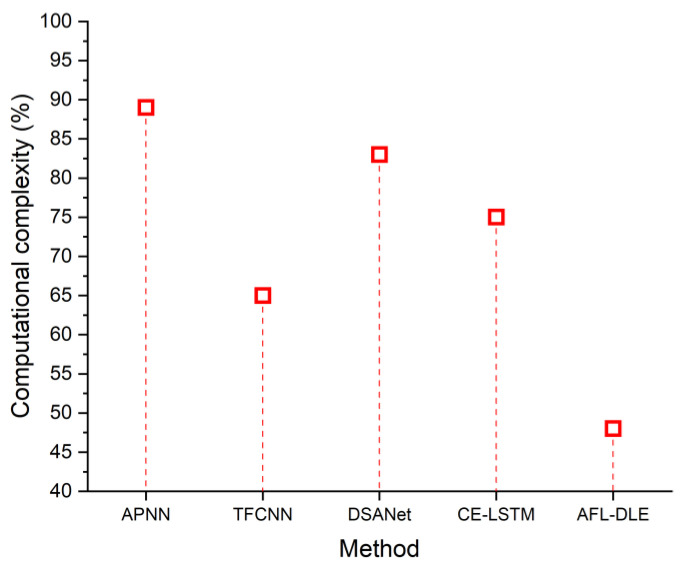
The computational complexity of the proposed and existing techniques, namely APNN [33], TFCNN [34], DSANet [35] and CE-LSTM [36].

Computational complexity measures the proportion of processing assets (work and area) that a certain algorithm consumes as it executes when it is being performed. When using a UVLC system, the computational complexity is enormous. Table 3 indicates the comparison of Computational complexity (%). The computational complexity of the suggested and existing approaches is shown in Figure 7. This demonstrates that the proposed method has a 48% lower level of computational complexity. The formula for computational complexity can be determined as follows:

(15) $$T\left(n\right)=c,\;S\left(n\right)=c$$

Here, *T*(*n*) denotes the runtime, *S*(*n*) indicates the space usage, and *c* is a constant.

**Table 3:** Comparison of computational complexity (%).

Source	Computational Complexity (%)
APNN [33]	89
TFCNN [34]	65
DSANet [35]	83
CE-LSTM [36]	75
AFL-DLE [Proposed]	48

### 5.4. Transmission Rate

**Figure 8:**
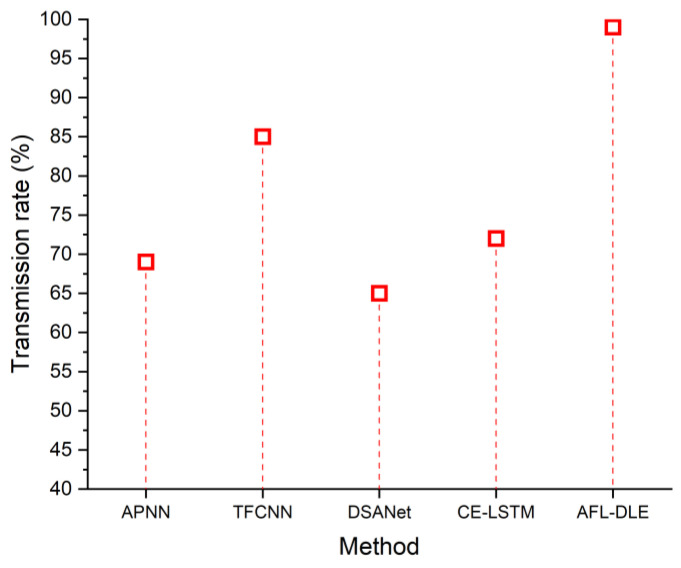
The transmission rate of the proposed and existing techniques, namely APNN [33], TFCNN [34], DSANet [35] and CE-LSTM [36].

The quantity of information that may be sent through a data terminal or a communication medium within a certain amount of time is referred to as the data transmission rate. Linear and nonlinear distortions impact the transmission rate in UVLC systems. A communication indication is a visual or audible cue that conveys a person’s status or level of communication readiness. It enables others to ascertain whether anyone has access to communication or if they are unavailable or otherwise engaged. In order to enhance successful communication and prevent needless disruptions, communication indicators are frequently employed in a variety of situations, including workplaces, businesses, and internet platforms. The transmission rate may be increased by removing the distortion. Table 4 shows the comparison of the transmission rate (%). Figure 8 displays the transmission rates of the proposed and existing methods. It demonstrates that the suggested approach has a 99% higher transmission rate. The transmission rate can be calculated as

(16) $$Transmission\;Rate=Channel\;Capacity\;*\;\left(1-Error\;Rate\right)$$

The data rate, which may be transmitted at its highest rate via a given communication channel, is known as channel capacity. It is dependent on the channel’s *SNR*, bandwidth, and other characteristics. The probability of a transmission error is known as the error rate and is normally expressed as a decimal value ranging from zero to one.

**Table 4:** Comparison of the transmission rate (%).

Source	Transmission Rate (%)
APNN [33]	69
TFCNN [34]	85
DSANet [35]	65
CE-LSTM [36]	72
AFL-DLE [Proposed]	99

### 5.5. Computation Cost

**Figure 9:**
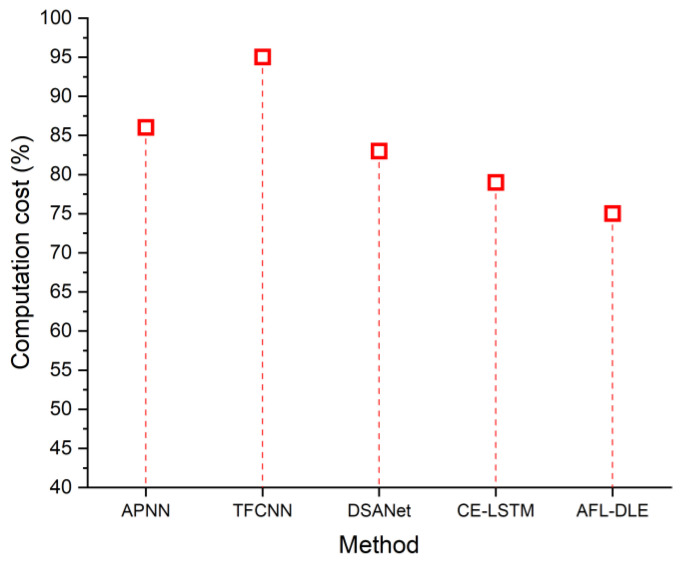
The computation cost of the proposed and existing techniques, namely APNN [33], TFCNN [34], DSANet [35] and CE-LSTM [36].

Money is saved when a technological component is enhanced over time to function more efficiently. To maximize profitability, the technology aims to raise overall effectiveness and reduce acquisition expenses. The computational cost of UVLC systems is often extremely high. Figure 9 displays the cost-effectiveness of the recommended and existing solutions. Table 5 shows the comparison of computation cost (%). The presently used techniques are 75% less expensive than the suggested alternatives. The following is the formula for computation cost:

(17) $$T\left(n\right)=O\left(f\left(n\right)\right)$$

*T*(*n*) denotes the algorithm’s time complexity, and *f*(*n*) is a function that gauges the procedure’s runtime’s development as input size rises.

**Table 5:** Comparison of computation cost (%).

Source	Computation Cost (%)
APNN [33]	86
TFCNN [34]	95
DSANet [35]	83
CE-LSTM [36]	79
AFL-DLE [Proposed]	75

### 5.6. Discussion

Chen et al. [33] suggested a computationally effective pre-distortion strategy premised on an “adaptive partitioning neural network (APNN)” to reduce nonlinear deficiencies in elevated UVLC technology. APNN is an acronym for adaptive partitioning neural networks. The UVLC mechanism becomes more complicated due to the distortions. Hence, this neural network is offered for the elimination of distortions. UVLC systems have employed deep neural networks to correct nonlinear deformation. In real implementations, it is crucial to prioritize the trade-off involving network intricacy and equilibrium effectiveness. In this study, Chen et al. [34] propose the use of a unique hybridized frequency response assisted “temporal convolutional neural network (TFCNN)” with attention strategy as the final equalization in a controlled UVLC network. The nonlinear distortions present in the UVLC technology may be removed using post-equalization. Yuan et al. [35] introduced a “Dual Self-Attention Network (DSANet)” as a final equalization for the CAP-controlled UVLC technology in their research. The effectiveness of the transmission is limited by the non-linearity influence introduced by the direct modulation/demodulation in UVLC. To solve this difficulty, Xu et al. [36] created the “convolution enhanced long short-term memory (CE-LSTM)” neural system, which was used to improve UVLC devices’ efficiency under severe non-linearity by overcoming linear and nonlinear distortion. The previous nonlinear post-equalizer using deep learning still has issues, such as how much data nodes affect the result, how much the equilibrium impact declines as the data flow increases, and how a model’s complexity affects how long it takes to train.

## 6. Conclusions

UVLC technology has seen a sharp increase in demand in recent years. This is mostly due to the fact that an increasing number of technological advancements are implementing their communication systems in underwater environments. The UVLC systems are affected by linear and nonlinear distortions, which are problematic. The outcome is that it makes the system less effective. Since the UVLC system exhibits linear and nonlinear distortion, we proposed an adaptive fuzzy logic deep-learning equalizer (AFL-DLE) to reduce both effects. The Enhanced Chaotic Sparrow Search Optimization Method (ECSSOA) is an algorithm that was created to boost system performance through optimization. The developed prototype technique considers the received signals’ distortion and dispersion. Following that, it divides the constellation’s signals into distinct segments, equalized by two differently sized sophisticated networks. The experimental results demonstrate that the recommended AFL-DLE was effective in reaching the bit error rate (55%) and distortion rate (45%), as well as reducing computational complexity (48%), improving transmission rate (99%), and lowering computation cost (75%). The recommended approach outperforms other methods, indicating that it is more effective when compared to them. Internet data processing can now be implemented in high-speed UVLC systems thanks to the AFL-DLE’s effectiveness and viability in real-world applications. In a prospective study, a uniformly distributed constellation stochastic structuring method will be investigated to enhance the performance of UVLC systems further.

## Data Availability

Not applicable.
